# Hereditary Angioedema Presenting With Acute Abdominal Symptoms and Progressive Upper Airway Compromise: A Case Report

**DOI:** 10.7759/cureus.117598

**Published:** 2026-10-07

**Authors:** Debasmita Mondal, Bhupeshwari Patel, Babu Lal

**Affiliations:** 1 Trauma and Emergency Medicine, All India Institute of Medical Sciences, Bhopal, Bhopal, IND

**Keywords:** acute abdomen, airway compromise, diagnostic challenge, fresh frozen plasma, hereditary angioedema

## Abstract

Hereditary angioedema (HAE) is a rare but potentially life-threatening disorder that may present with abdominal symptoms and airway compromise, often mimicking surgical or allergic emergencies. We report the case of a 21-year-old female patient who presented to the emergency department with severe abdominal pain, vomiting, and abdominal distension, followed by progressive facial and tongue swelling, muffled voice, and inspiratory stridor during observation. Initial laboratory testing showed no leukocytosis, electrolyte disturbance, or acid-base abnormality, while plain abdominal radiography showed no free intraperitoneal air or obstructive bowel gas pattern. The patient showed no clinical improvement with conventional anaphylaxis management, including epinephrine, corticosteroids, and antihistamines. A history of recurrent abdominal pain episodes, family history of swelling episodes, absence of urticaria or hypotension, and evolving angioedema prompted consideration of hereditary angioedema. Because disease-specific therapy was unavailable, two units of fresh frozen plasma were administered, followed by improvement in abdominal and upper airway symptoms. Endotracheal intubation and surgical intervention were not required. Following stabilization, low serum C4 and C1 esterase inhibitor levels supported the diagnosis of hereditary angioedema type I. This case highlights the diagnostic challenges associated with atypical presentations of hereditary angioedema and emphasizes the importance of early recognition, airway vigilance, and resource-appropriate management in emergency settings.

## Introduction

Hereditary angioedema (HAE) is a rare autosomal dominant disorder with an estimated prevalence of approximately one in 50,000 individuals, characterized by recurrent episodes of non-pitting edema involving the skin, gastrointestinal tract, and upper airway (Online Mendelian Inheritance in Man or OMIM #106100) [1,2]. The disease is most commonly caused by quantitative or functional deficiency of C1 esterase inhibitor (C1-INH), resulting in excessive bradykinin-mediated vascular permeability and episodic angioedema [1-2]. HAE with C1-INH deficiency (HAE-C1-INH), historically classified as type I (quantitative deficiency) and type II (functional deficiency), is caused by pathogenic variants in the serpin family G member 1 (SERPING1) gene encoding C1-INH.

In addition, HAE with normal C1-INH (HAE-nC1-INH) comprises genetically distinct forms associated with pathogenic variants in genes including coagulation factor XII (F12), plasminogen (PLG), angiopoietin 1 (ANGPT1), kininogen 1 (KNG1), myoferlin (MYOF), and heparan sulfate-glucosamine 3-O-sulfotransferase 6 (HS3ST6), as well as forms in which the underlying genetic cause remains unidentified [3,4]. Although cutaneous swelling is the most recognized manifestation, gastrointestinal involvement may predominate in some patients and present with severe abdominal pain, vomiting, abdominal distension, and ascites, frequently mimicking acute surgical emergencies [2,5,6].

Early recognition of HAE in emergency settings remains challenging because many patients initially present without a prior diagnosis and may be misdiagnosed as having allergic angioedema, anaphylaxis, or an acute surgical process [5-7]. Unlike histamine-mediated angioedema, HAE typically does not respond to epinephrine, antihistamines, or corticosteroids, making early diagnostic suspicion particularly important in patients with progressive airway involvement [1,2]. Current international guidelines recommend early on-demand treatment of acute HAE attacks, with intravenous C1-INH, icatibant, or sebetralstat recommended as first-line therapies [3,4]. Delayed recognition may result in unnecessary interventions, repeated hospitalizations, and potentially fatal airway compromise [7].

We present a previously undiagnosed young adult with recurrent abdominal attacks who developed progressive upper airway edema during emergency department observation. The case is notable for the combination of an acute abdominal presentation followed by evolving life-threatening airway involvement and clinical improvement after fresh frozen plasma administration when recommended on-demand HAE therapies were unavailable. This case highlights the diagnostic distinction between bradykinin-mediated angioedema and anaphylaxis, as well as the challenges of airway preparedness and alternative treatment in a resource-constrained emergency setting.

## Case presentation

A 21-year-old woman with no significant past medical history presented to the emergency department with severe diffuse abdominal pain of six-hour duration, associated with abdominal distension and three episodes of non-bilious, non-bloody vomiting. There was no history of fever, diarrhea, dysuria, recent drug intake, insect bite, trauma, or known food allergy.

About 30 minutes after presentation, while being evaluated in the emergency department, she developed progressive swelling of the lips, cheeks, and tongue, with a muffled voice and inspiratory stridor, indicating worsening upper airway involvement. She had no drooling, dysphagia, difficulty handling secretions, floor-of-mouth swelling, uvular or soft-palate edema, or reduced mouth opening. The swelling gradually progressed over the following hour. About 10 minutes after the swelling began, she developed a non-pruritic erythematous rash on the chest and upper limbs. There was no urticaria, wheezing, hypotension, or syncope.

The patient reported multiple previous episodes of unexplained abdominal pain that had resolved spontaneously without medical evaluation. No specific precipitating factors were identified in relation to these episodes. Her father had a history of recurrent, self-resolving episodes of facial swelling; however, he had never undergone formal evaluation or genetic testing. No similar history was reported in other family members.

On examination, the patient was conscious but anxious. Her heart rate was 110 beats/minute (reference range: 60-100 beats/minute), blood pressure 108/70 mmHg (normal adult range approximately 90/60 to <120/80 mmHg), respiratory rate 22 breaths/minute (reference range: 12-20 breaths/minute), and oxygen saturation 95% on room air (reference range: 95-100%); she was afebrile. Facial erythema and visible lip swelling were present. Dermatological examination revealed a transient, non-pruritic, non-blanching petechial eruption over the chest and upper limbs. Abdominal examination demonstrated diffuse tenderness, abdominal distension, and sluggish bowel sounds, without guarding, rigidity, or rebound tenderness. Respiratory examination revealed progressive facial and tongue edema with muffled voice and inspiratory stridor, while bilateral air entry remained preserved without wheeze or crepitations.

At the time of initial emergency department assessment, laboratory investigations showed hemoglobin of 12 g/dL, total leukocyte count of 9,800/mm³, and normal serum electrolytes. The investigations have been summarized in Table 1.

**Table 1:** Summary of investigations *Performed during subsequent evaluation after stabilization in the emergency department. ABG: Arterial Blood Gas; PaCO₂: Partial Pressure of Carbon Dioxide; PaO₂: Partial Pressure of Oxygen.

Investigation	Result	Reference Range	Interpretation
Hemoglobin	12.0 g/dL	12–15 g/dL	Within normal limits
Total leukocyte count	9,800/mm³	4,000–11,000/mm³	No leukocytosis
ABG pH	7.40	7.35–7.45	Normal acid-base status
PaCO₂	39 mmHg	35–45 mmHg	Normal
PaO₂	92 mmHg	80–100 mmHg	Normal oxygenation
Serum bicarbonate (HCO₃⁻)	24 mmol/L	22–28 mmol/L	Normal
Serum sodium (Na⁺)	138 mmol/L	135–145 mmol/L	Normal
Serum potassium (K⁺)	4.1 mmol/L	3.5–5.0 mmol/L	Normal
Serum chloride (Cl⁻)	102 mmol/L	98–106 mmol/L	Normal
Serum C4 level*	6 mg/dL	10–40 mg/dL	Reduced complement level
C1 esterase inhibitor level*	7 mg/dL	21–39 mg/dL	Consistent with hereditary angioedema type I

Electrocardiography (Figure 1) demonstrated sinus tachycardia.

**Figure 1:**
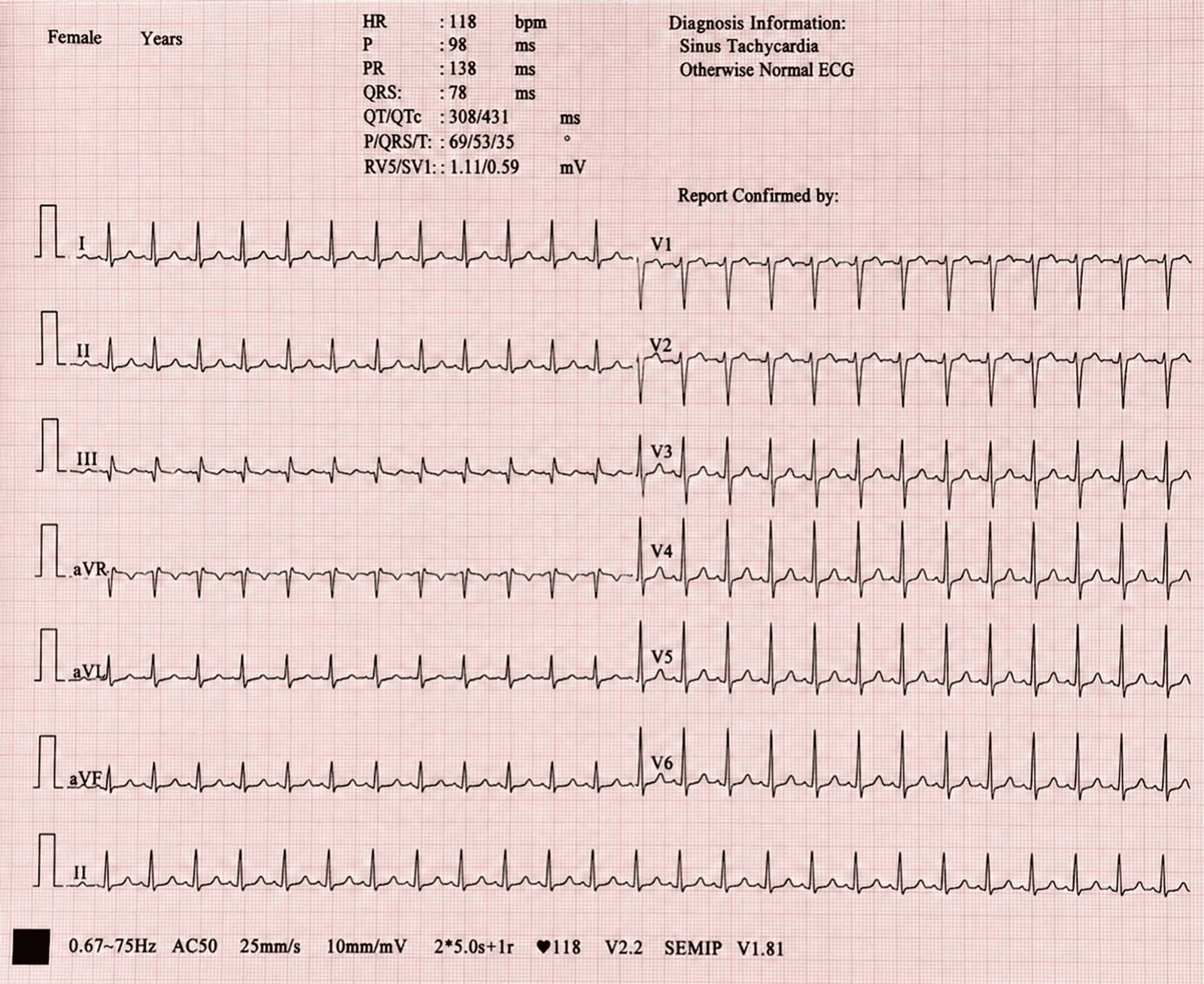
Electrocardiogram obtained at presentation Twelve-lead electrocardiogram demonstrating sinus tachycardia without evidence of acute ischemic changes, conduction abnormalities, or significant arrhythmias (measurements obtained at a different time than that mentioned in the text).

Chest radiography (Figure 2) showed no acute abnormality.

**Figure 2:**
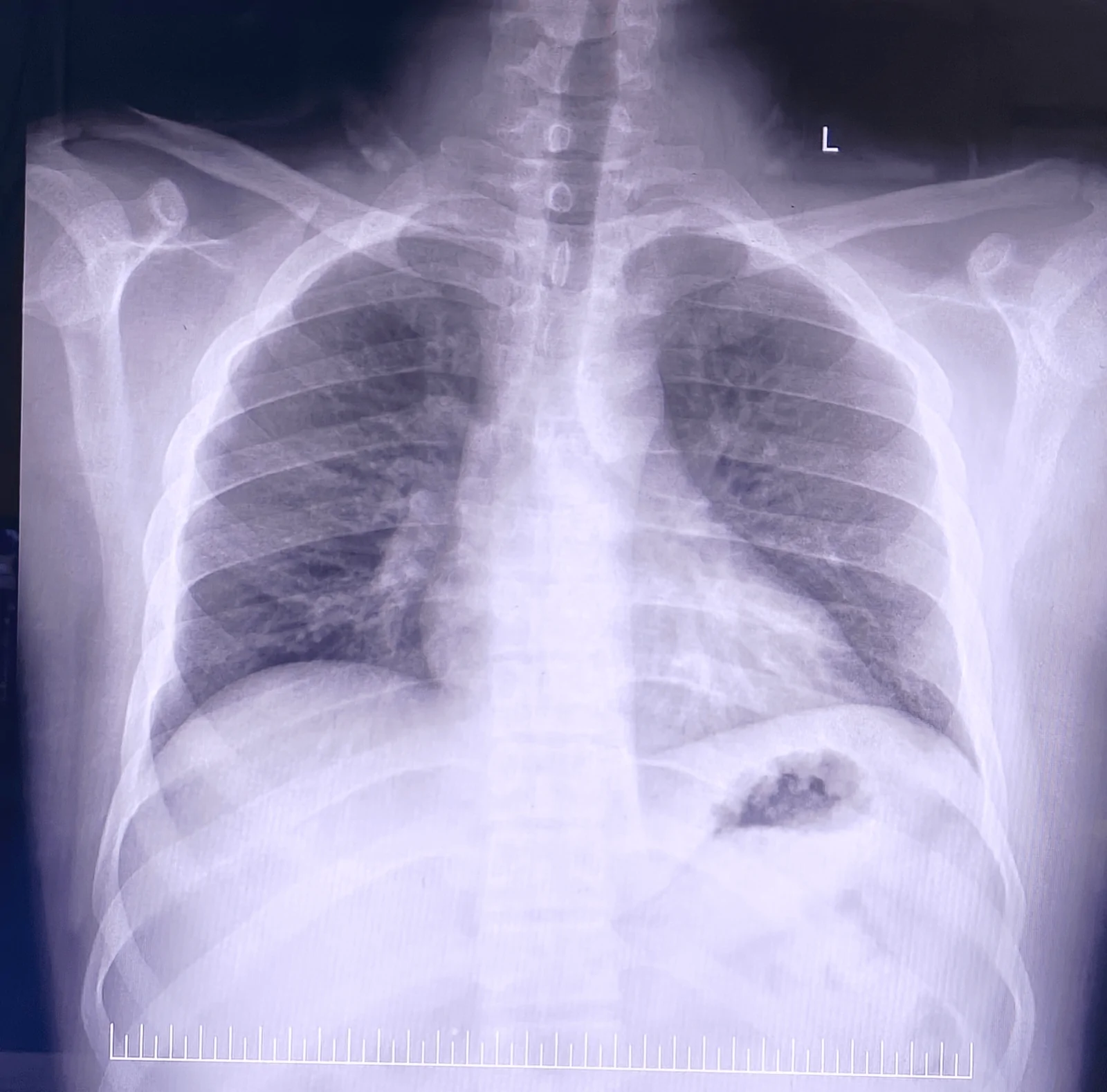
Chest radiograph at the initial presentation Chest radiograph showing no acute cardiopulmonary abnormality. There is no evidence of pneumothorax, pleural effusion, pneumomediastinum, or other acute thoracic pathology.

Plain abdominal radiography (Figure 3) demonstrated a non-obstructive bowel gas pattern, with no free intraperitoneal air or air-fluid levels.

**Figure 3:**
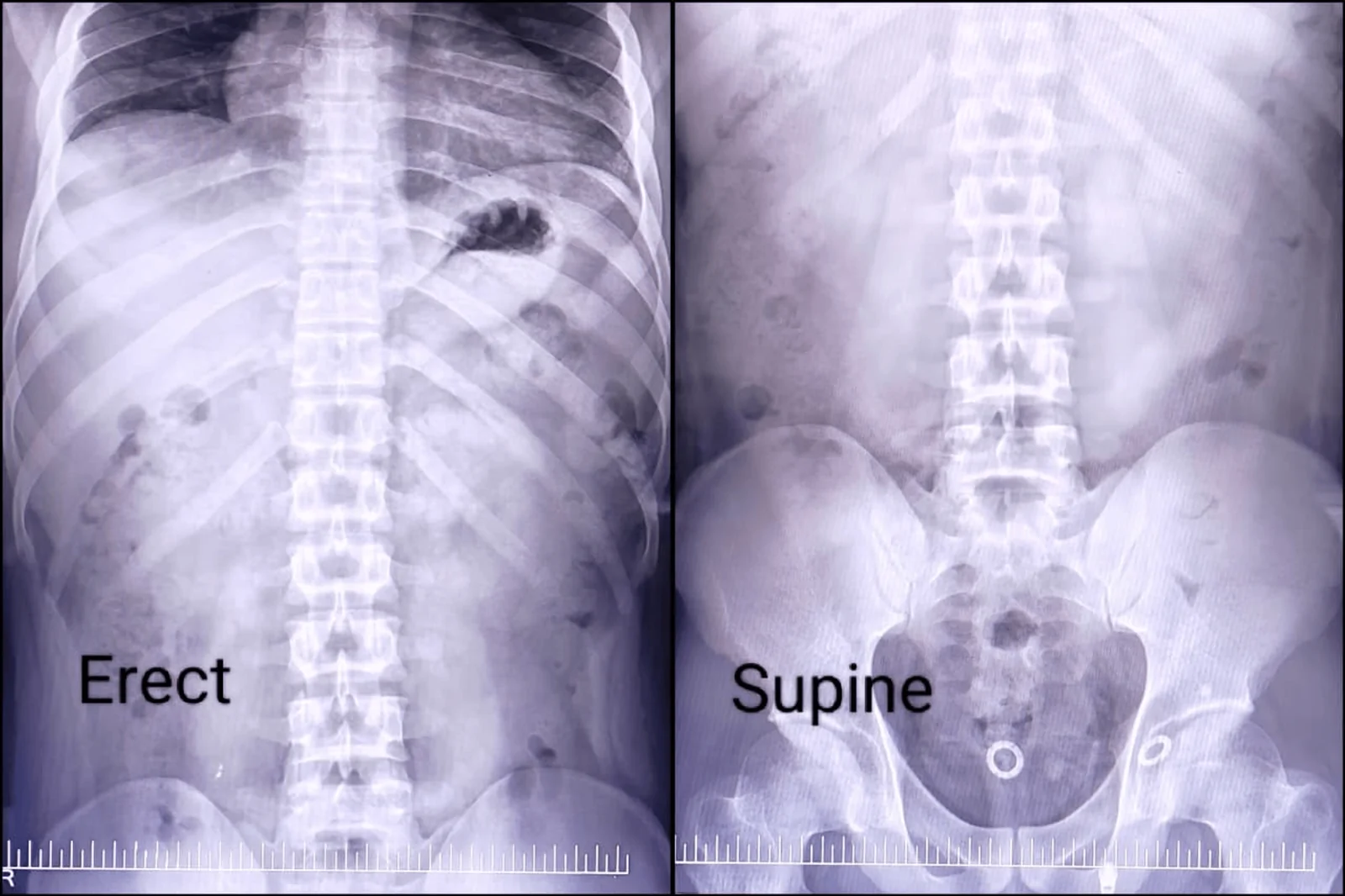
Plain abdominal radiograph at the initial presentation Erect and supine abdominal radiograph demonstrating a non-obstructive bowel gas pattern with no evidence of bowel obstruction, free intraperitoneal air, or abnormal air-fluid levels.

Given the combination of progressive upper airway swelling and erythematous rash, anaphylaxis was initially suspected. Approximately five minutes after the onset of the rash, corresponding to approximately 15 minutes after the onset of airway swelling, the patient received adrenaline 0.5 mg intramuscularly, two doses five minutes apart, pheniramine maleate 45.5 mg intramuscularly, and hydrocortisone 100 mg intravenously. On reassessment following this treatment, there was no clinical improvement.

Because of the progressive upper airway involvement, a difficult-airway cart and surgical airway tray were kept immediately available, and the patient was maintained in an upright position with supplemental oxygen at 6 L/min via face mask. The patient was managed by the emergency physicians with continuous airway, respiratory, and hemodynamic monitoring. Despite the ongoing upper airway edema, she remained clinically stable without evidence of worsening respiratory distress or hypoxemia; therefore, immediate airway intervention was deferred. Airway findings were reassessed at frequent intervals, with endotracheal intubation or surgical airway intervention planned in the event of clinical deterioration or evidence of impending airway compromise.

The differential diagnosis included acute abdominal pathology, allergic angioedema or anaphylaxis, and hereditary angioedema. Perforation and mechanical bowel obstruction were considered less likely because there were no peritoneal signs, and plain abdominal radiography showed no free intraperitoneal air or obstructive bowel gas pattern. Other intra-abdominal causes could not be definitively excluded by plain radiography alone. Although the presence of rash and airway involvement initially suggested allergic angioedema, the absence of urticaria, hypotension, wheeze, pruritus, or identifiable allergen exposure, along with failure to respond to conventional anaphylaxis treatment, argued against this diagnosis. The history of recurrent abdominal pain episodes, family history of swelling episodes, and evolving airway edema raised strong clinical suspicion for hereditary angioedema.

Approximately one hour after the onset of facial and tongue swelling, disease-specific therapies, including C1-INH concentrate, icatibant, and ecallantide, remained unavailable. After consideration of the potential risks and limited evidence supporting its use in HAE, fresh frozen plasma was arranged as an alternative treatment, and two units were administered intravenously over approximately one hour following appropriate consent. Supportive management, including intravenous fluids, continuous pulse oximetry, close hemodynamic monitoring, and standby airway support, was continued throughout. No transfusion-related adverse events were observed.

Within approximately one hour of fresh frozen plasma administration, the patient's abdominal pain improved, the progression of airway edema stabilized, and her voice improved. Approximately two hours later, the erythematous rash had completely resolved, facial and tongue swelling had substantially decreased, and inspiratory stridor had disappeared. Her oxygen saturation improved to 99% on room air. Endotracheal intubation or surgical airway intervention was not required.

The patient remained clinically stable during 24 hours of observation and was subsequently discharged with advice to seek early medical attention for recurrent abdominal symptoms or any features of upper airway swelling, particularly given the potential for rapid progression and airway compromise. As no specific precipitating trigger had been identified, no specific trigger avoidance advice was provided. Follow-up with the immunology department was advised.

During subsequent outpatient evaluation, a blood sample for complement testing that had been collected before fresh frozen plasma administration was analyzed, with results becoming available approximately one week later. Serum C4 was 6 mg/dL (reference range, 10-40 mg/dL) and C1-INH antigen was 7 mg/dL (reference range, 21-39 mg/dL), supporting a diagnosis of hereditary angioedema type I in the context of recurrent abdominal symptoms and a positive family history. Functional C1-INH was not performed, and repeat complement testing after recovery from the acute episode was not available. Genetic testing, including SERPING1 sequencing, was considered given the positive family history but could not be performed because of financial constraints. Extended longitudinal follow-up, including subsequent recurrence of abdominal or airway symptoms, access to disease-specific on-demand therapy, consideration of long-term prophylaxis, and formal family screening, was not available.

The chronological sequence of symptom progression, treatment, monitoring, and clinical response is summarized in Table 2

**Table 2:** Chronological timeline of clinical presentation, management, and outcome The table summarizes the chronological progression of symptoms, clinical findings, emergency department management, response to treatment with fresh frozen plasma (FFP), and subsequent outcome in a patient with hereditary angioedema (HAE). Time is expressed relative to emergency department (ED) presentation (0 h), with −6 h indicating symptom onset before presentation. C1-INH: C1 esterase inhibitor; IM: intramuscular; IV: intravenous; SpO₂: peripheral oxygen saturation.

Time	Clinical event
−6 h	Onset of severe diffuse abdominal pain
0 h	ED presentation with abdominal pain, distension, and vomiting
+30 min	Progressive swelling of lips, cheeks, and tongue with muffled voice and mild inspiratory stridor
+40 min	Non-pruritic erythematous rash developed over chest and upper limbs
+45 min	Adrenaline 0.5 mg IM × 2 doses, pheniramine maleate 45.5 mg IM, and hydrocortisone 100 mg IV administered
Following treatment	No clinical improvement; continuous vital-sign and airway monitoring maintained; difficult-airway and surgical-airway equipment kept ready
~+1 h 30 min	Approximately 1 hour after onset of airway swelling: FFP arranged because disease-specific HAE therapies were unavailable
Subsequently	Two units of FFP administered IV over approximately 1 hour
Within ~1 h of FFP administration	Abdominal pain improved; airway edema stabilized; voice improved
~2 h later	Rash resolved; facial/tongue swelling substantially decreased; stridor disappeared; SpO₂ 99% on room air
Next 24 h	Clinically stable under observation; no intubation or surgical airway required
Subsequent outpatient evaluation	Low C4 and C1-INH levels supported HAE type I

## Discussion

Abdominal involvement is a well-recognized manifestation of HAE and may mimic an acute surgical abdomen. Previously reported cases have described isolated gastrointestinal involvement, including bowel wall edema, abdominal pain, vomiting, and diarrhea. Marrawani et al. reported a 24-year-old woman with HAE who presented with severe abdominal symptoms and isolated ascending and transverse colon involvement, mimicking an acute abdomen. However, that patient had a known history of recurrent angioedema and a previous episode of airway compromise requiring emergency intubation [5]. In contrast, our patient had no previous diagnosis of HAE and presented initially with isolated severe abdominal symptoms before developing progressive facial and tongue swelling with inspiratory stridor during emergency department observation. Thus, the evolution from an apparently gastrointestinal presentation to clinically significant upper airway involvement within the same acute episode represents an important feature of the present case.

The diagnostic relevance of family history is also illustrated in previously reported HAE cases. Xia et al. described two pediatric HAE families in which recurrent abdominal or peripheral swelling was associated with a positive family history, and molecular testing identified SERPING1 variants [8]. Their literature review further demonstrated that abdominal pain and upper respiratory tract edema are both important manifestations of pediatric HAE [8]. Similarly, Widhani et al. described a patient with longstanding recurrent angioedema and multiple affected family members, in whom diagnosis was complicated by limited access to laboratory testing and HAE-specific treatment [9]. In our patient, the combination of recurrent unexplained abdominal pain and a father with recurrent facial swelling provided important retrospective clues to an inherited disorder, although neither the patient nor her father had previously undergone formal evaluation. The absence of an established diagnosis at presentation increased the initial diagnostic uncertainty, particularly because the subsequent petechial eruption and upper airway edema prompted consideration of anaphylaxis. Physical exertion has been described as a potential precipitating factor for HAE attacks; however, no specific precipitating factor was identified in the present case [10].

Management in the present case was additionally influenced by limited access to disease-specific therapy. Current international guidelines recommend early on-demand treatment of acute HAE attacks with intravenous C1-INH, icatibant, or sebetralstat, with other therapies considered when first-line options are unavailable or infeasible [3,4]. In our setting, C1-INH concentrate, icatibant, and ecallantide were unavailable. Fresh frozen plasma was therefore administered as an alternative treatment in the setting of progressive upper airway involvement. Similar treatment limitations have been described in resource-constrained settings, including the report by Widhani et al., in which access to first-line HAE therapies was limited [9,11-13]. In the present case, clinical improvement followed fresh frozen plasma administration; however, given the single-case nature of the observation and the temporal association alone, this response should not be interpreted as establishing a causal treatment effect. Fresh frozen plasma should therefore be considered an alternative option when recommended disease-specific therapies are unavailable, rather than an equivalent substitute for targeted treatment.

Airway assessment and preparedness remain critical in patients with suspected HAE because progressive tongue, pharyngeal, and laryngeal edema may worsen rapidly, distort airway anatomy, and make airway management increasingly difficult [1,2]. Early recognition of progressive airway compromise and timely consideration of airway intervention are therefore essential. In the present case, early anticipation of airway compromise allowed preparation for advanced airway management while definitive treatment was initiated. Current international guidelines recommend early on-demand treatment of HAE attacks, with intravenous C1-INH, icatibant, or sebetralstat as first-line therapies [3,4]. Ecallantide may be considered when first-line therapies are unavailable or infeasible, while solvent detergent-treated plasma or fresh frozen plasma may be considered when targeted therapies are unavailable [4,11,12]. Fresh frozen plasma is therefore an alternative rather than an equivalent to first-line targeted therapy and may be particularly relevant in resource-constrained settings where access to disease-specific agents is limited. Its use is nevertheless limited by the theoretical potential for worsening angioedema through additional complement components, as well as the risks of transfusion reactions and transmission of blood-borne infections [4]. In the present case, targeted therapies were unavailable, and two units of fresh frozen plasma were administered, followed by improvement in abdominal pain, facial and tongue swelling, voice quality, and stridor. Endotracheal intubation and surgical intervention were not required. Given the temporal association in this single case, the clinical improvement should not be interpreted as establishing a causal effect of fresh frozen plasma.

This case has several limitations. First, as a single case report, the findings cannot establish the generalizability of the clinical course or the efficacy of fresh frozen plasma. The severity of airway involvement was assessed clinically and was not objectively graded, and direct laryngeal visualization was not performed. Abdominal cross-sectional imaging was not performed, limiting further characterization of gastrointestinal involvement and exclusion of other intra-abdominal pathology. Although complement testing was performed on a blood sample obtained before fresh frozen plasma administration, functional C1-INH activity was not assessed and repeat complement testing after recovery from the acute episode was not available. Thus, the available biochemical findings supported the diagnosis in the context of the clinical presentation and positive family history but did not provide functional or repeat biochemical confirmation. Finally, the temporal association between fresh frozen plasma administration and clinical improvement in this single case cannot establish treatment efficacy. Extended longitudinal follow-up was also unavailable to assess subsequent recurrence, long-term prophylaxis, or access to disease-specific on-demand therapy.

This case further emphasizes the pivotal role of emergency physicians in recognizing atypical presentations of hereditary angioedema. Early pattern recognition, appropriate clinical assessment of potential surgical emergencies, and prompt institution of resource-appropriate therapy are essential to reduce morbidity and mortality associated with this condition. Long-term management of hereditary angioedema includes specialist follow-up, assessment for the need for long-term prophylaxis, access to effective on-demand therapy, and patient education regarding recognition of early symptoms and potential triggers. Genetic counseling and evaluation of first-degree relatives may also be considered because of the hereditary nature of the disorder [3,4,13,14]. Patients should be provided with an individualized emergency action plan and advised to seek urgent medical attention for symptoms suggestive of upper airway involvement. In the present case, immunology follow-up was advised, although longer-term follow-up and access to disease-specific on-demand or prophylactic therapy could not be assessed.

## Conclusions

HAE should be considered in patients with recurrent abdominal symptoms followed by angioedema without urticaria, particularly when symptoms do not respond to standard anaphylaxis treatment. Progressive tongue swelling, voice change, or stridor requires early airway planning and close monitoring. When recommended on-demand therapies are unavailable, fresh frozen plasma may be considered as a fallback option; however, the clinical improvement observed in this single case represents a temporal association and should not be interpreted as evidence of treatment efficacy or as demonstrating that fresh frozen plasma prevented the need for airway or surgical intervention.
